# MiR-199b-5p Promotes Gastric Cancer Progression by Regulating HHIP Expression

**DOI:** 10.3389/fonc.2021.728393

**Published:** 2021-08-31

**Authors:** Songda Chen, Huijie Wu, Lingyu Zhu, Mengjie Jiang, Shuli Wei, Jinhua Luo, Aiqun Liu

**Affiliations:** ^1^Department of Endoscopy, Guangxi Medical University Cancer Hospital, Nanning, China; ^2^Department of Gastroenterology, The 10th Affiliated Hospital of Guangxi Medical University, Qinzhou, China

**Keywords:** miR-199b-5p, *HHIP*, GC, bioinformatics, maker

## Abstract

**Objectives:**

Gastric cancer (GC) is one of the most common malignant tumors. More and more evidences support the role of microRNAs (miRNAs) in tumor progression. However, the role of miRNAs in human GC remains largely unknown.

**Methods:**

Based on the published gastric cancer expression profile data, combined with bioinformatics analysis, potential miRNAs in the process of GC were screened. The expression of miR-199b-5p in GC cells and patients’ plasma was detected by RT-PCR. The effects of miR-199b-5p on GC *in vitro* were detected by EdU proliferation assay, colony formation assay, Transwell assay and wound healing assay. Western blot was used to detect epithelial-mesenchymal transition (EMT) related proteins. The subcutaneous tumorigenesis model and metastatic tumor model of mice were used to study its effect *in vivo*. Bioinformatics and Dual luciferase reporter assay were used to verify the effect of miR-199b-5p and its target gene.

**Results:**

Through bioinformatics analysis, we screened a novel miRNA miR-199b-5p that was significantly up-regulated in GC tissue and associated with poor prognosis of GC patients. RT-PCR results showed that its expression was also up-regulated in GC cell lines and patients’ plasma. MiR-199b-5p can significantly promote GC cell proliferation and migration *in vitro* and *in vivo*. Western blot showed that miR-199b-5p could promote the EMT process of GC. HHIP has been proved to be a target of miR-199b-5p, and the recovery of HHIP can weaken the effect of miR-199b-5p.

**Conclusion:**

MiR-199b-5p may play an oncogene role in GC by targeting *HHIP*, suggesting that miR-199b-5p may be a potential therapeutic target for GC.

## Introduction

Although the incidence of gastric cancer (GC) has declined in the past few decades, it is still one of the most common malignant tumors, especially in East Asia, where its incidence and mortality rank fourth and third in the world, respectively (1, 2). GC patients are often in the advanced stage after diagnosis, and one of the reasons for their high mortality is the lack of specific diagnosis. Although early diagnosis, surgical techniques, and postoperative radiotherapy and chemotherapy have gradually improved the clinical prognosis of GC, the 5-year survival rate of patients with advanced GC is still relatively low (3).The poor prognosis of patients with advanced GC is mainly attributed to invasion and metastasis, among which lymph node metastasis exceeds 50% (4). Therefore, it is extremely necessary to explore the molecular mechanism of GC progression, which may provide a basis for new therapeutic targets for GC.

miRNA is a type of non-coding RNA composed of 20-24 nucleotides, which plays an important role in human health and disease by regulating gene expression (5, 6). MiRNA was first discovered in 1993, and it regulates more than 50% of the known genes in humans (7, 8). It has been reported that miRNAs can have multiple target genes, and multiple miRNAs can also regulate the same target gene (9). Mature miRNAs can play a bidirectional regulatory function by degrading or inhibiting the translation of target mRNAs, that is, miRNAs actually assume at least two functions of oncogene or tumor suppressor gene in life activities. Study has found that miR-199b-5p is highly expressed in osteosarcoma and promotes the malignant development of osteosarcoma (10). However, miR-199b-5p is under-expressed in prostate cancer and breast cancer and inhibits tumor cell growth (11, 12). These studies show that miR-199b-5p plays a two-way regulatory role in different cancer cells. However, the related functions of miR-199b-5p in GC have not been reported yet. Through bioinformatics, we found that miR-199b-5p is significantly highly expressed in gastric cancer tissues, and the highly expressed miR-199b is related to the poor prognosis of gastric cancer patients. Therefore, we were very interested in the role of miR-199b-5p produced from the 5,arm in gastric cancer. In this study, we found that miR-199b-5p can promote the progress of GC both *in vivo* and *in vitro*.

Hedgehog interacting protein (*HHIP*) gene is located on chromosome 4q31.21–31.3 and encodes the production of *HHIP*. As an evolutionarily conserved protein, HHIP is a key mediator of many basic processes in embryonic development (13). *HHIP* is an endogenous antagonist of the hedgehog signaling pathway, and its loss of function or mutation may lead to the up-regulation of this signal and promote tumorigenesis (14, 15). Scholars have found that the expression of *HHIP* in gastric cancer tissues is reduced, and the overexpression of *HHIP* reduces the migration and invasion of GC (16). We have confirmed that *HHIP* is the direct target of miR-199b-5p and plays an important role in human GC through bioinformatics analysis and related functional analysis.

In this study, we aimed to explore the biological role of miR-199b-5p and its relationship with *HHIP*. Our research results show that over-expression of miR-199b-5p can promote the progress of GC *in vivo* and *in vitro*, and on the contrary, it can inhibit the malignant development of GC. *HHIP* is the direct target gene of miR-199b-5p. Overexpression of HHIP can partially reverse the role of miR-199b-5p in GC, that is, the related role of miR-199b-5p in GC was partly caused by regulating *HHIP*. These findings also provided a basis for miR-199b-5p as a potential therapeutic target for GC.

## Materials and Methods

### Microarray Data

Three GC datasets (GSE93415, GSE78091,GSE23739) were downloaded from GEO database. Among them, GSE93415 includes 20 pairs of GC tissues and 20 pairs of normal tissues, GSE78091 includes 3 pairs of GC tissues and 3 pairs of normal tissues, and GSE23739 includes 40 pairs of GC tissues and 40 pairs of normal tissues. *P* < 0.05 and [logFC] > 1 as screening conditions

### Tissue Samples and GC Cell Lines

The specimens were collected in the Department of Pathology, Affiliated Guangxi Medical University Cancer Hospital. No radiotherapy or chemotherapy was performed before the operation, and the patients or their relatives signed informed consent form. This study was approved by the Ethics Committee of the Guangxi Medical University Cancer Hospital. Human GC cell lines, AGS, MGC803, SGC7901 and rumen-free epithelial cells GES1 were purchased from the Shanghai Institute of Biological Sciences Center. All cell lines were cultured in DMEM medium (Gibico, USA) supplemented with 10% fetal bovine serum (Gibico, USA) and 1% penicillin/streptomycin (Gibico, USA) in a cell incubator at 37°C and 5% CO _2_.

### RNA Extraction and PCR

Total RNA was extracted from GC cells using TRIzol reagent (Beyotime, China), according to the manufacturer’s agreement. U6 and β-actin were used as endogenous controls to quantify miRNA and mRNA, respectively. All primers(Shangon Biotech, China) used in our research are as follows: β-actin forward, 5, -GCATCGTCACCAACTGGGAC-3, andβ-actin reverse, 5, -ACCTGGCCGTCAGGCAGCTC-3,; U6 forward, 5’-CTCGCTTCGGCAGCACA-3’ and U6 reverse, 5’-AACGCTTCACGAATTTGCGT-3’; HHIP forward, 5’-TCTCAAAGCCTGTTCCACTCA-3’ and HHIP reverse 5’-GCCTCGGCAAGTGTAAAAGAA-3’; miR-199b-5p:5’-CCCAGTGTTTAGACTATCTGT T-3’. The relative expression is quantified by the 2 ^−ΔΔC T^ method. Each sample was repeated three times for PCR.

### Cell Transfection

According to the experimental design, commercial lentiviral vectors (GenePharma, China)were used to construct LV-hsa-miR-199b-5p-mimic vector (miR-mimic), LV-miR-199b-5p-inhibitor vector (miR-inhibitor) and Lv-HHIP vector, and then cell transfection was carried out according to the reagent instructions. Finally, 5μg/mL puromycin (Beyotime, China) was used for about a week to obtain stable transfected cell lines.

### Colony Formation Assay

Stably transfected GC cells were placed in a 6-well plate (500 cells/well) and cultured in DMEM medium for about 2 weeks。The colonies were stained with 0.1% crystal violet (Beyotime, China) after washing away with PBS. All procedures were performed in triplicate.

### 5-Ethynyl-2-Deoxyuridine (EdU) Assay

EdU assay kit (RiboBio, China) was used to detect cell proliferation. First, the cells were seeded into a 96-well plate (2 × 10^4^cells/well) and cultured in complete medium for 24 hours. On the second day, cells were incubated with 50μM EdU about 2h at 37°C and fixed in 4% formaldehyde for 20min. And then 0.5% TritonX‐100 was permeabilized for 10 minutes at room temperature. After washing with PBS, 200μL 1×ApolloR reaction cocktail was added to react with the EdU for 30 minutes. Then add 200μL hoechst33342 for 10min to observe the nucleus. Images of cells were captured under a fluorescence microscope (Olympus Corp, Japan).

### Migration Assay

The migratory ability of cells were assayed by using a 6.5mm chamber with 8μm pores (Corning, USA). 2×10^4^ stably transfected GC cells were suspended in 200μL serum-free DMEM medium and placed on the top of the chamber, and then complete medium (500μL)was added into lower chamber. After the cells were cultured in a 37°C incubator for 24 hours, the cells in the upper layer of the chamber were removed with cotton swabs. Staining with 1% crystal violet for 30min after washing with PBS. We imaged and counted the cells on the bottom surface of membrane by microscope (Olympus Corp. Japan). All procedures were performed in triplicate.

### Wound Healing Assay

Stably transfected GC cells (5 × 10^5^) were seeded in six-well plates. After the cells adhere to the wall, 200μL sterile pipette tips were used to form linear scratches. Then the plate was washed several times with PBS to remove the suspended cells, and the cells were cultured in serum-free medium. After 0 and 24h, we imaged the wounds at the same position under the microscope (Olympus Corp. Japan) and the distance between the wound sides was calculated. All procedures were performed in triplicate.

### Western Blot Assay

Proteins were extracted from GC cells with RIPA lysis buffer (Beyotime, China), separated by sodium dodecyl sulfate polyacrylamide gel electrophoresis (SDS-PAGE), and then transferred to polyvinylidene fluoride (PVDF) membrane. The PVDF membrane was blocked with 5% skim milk at room temperature for 1.5 hours, and incubated with specific antibodies overnight at 4°C. On the second day, the membrane was incubated with the secondary antibody for 1 hour at room temperature. After washing with TBST, proteins were detected with enhanced chemiluminescence (ECL) detection system. β-actin was used as an internal control.

### Cell Immunofluorescence Assay

Cells in each group were sliced in six-well plate. The cells were fixed with 4% paraformaldehyde for 15 minutes and then sealed with 5% BSA at room temperature for 1 hour. Then, the primary antibody HHIP (affinity. USA) was dripped and incubated overnight at 4°C. After cleaning with PBS, Cy3 labeled secondary antibody (beyotime, China) was dropped and incubated at room temperature for 1 hour. Then the cell nucleus were labeled with Hoechst 33342. Finally, the film was sealed with anti-fluorescence quenching agent and observed with fluorescence microscope immediately.

### Immunohistochemistry

All samples were fixed with 4% paraformaldehyde solution and embedded in paraffin. Then, paraffin embedded sections were dewaxed in xylene and then rehydrated in graded ethanol. After antigen repair, endogenous catalase was blocked by 3% hydrogen peroxide. Then the primary antibody for HHIP (affinity. USA) and Ki-67 (Servicebio,China)was incubated on the slices overnight at 4°C. After PBS washing, the sections were incubated with HRP-polymer-conjugated secondary antibody at 37°C for 1 h, and then stained with 3,3-diaminobenzidine (DAB) solution for 3min. The nucleus were stained with hematoxylin. We randomly selected three observation fields to observe.

### Animal Experiment

4-week-old female BALB/c nude mice were purchased from the animal center of Guangxi Medical University. The experimental animals all conform to the regulations of the animal nursing and use Committee of Guangxi Medical University. In the subcutaneous tumorigenesis experiment, 15 female nude mice were randomly divided into three groups. The stably transfected cells (2 × 10^6^ cells/200μL PBS) were injected into the lateral abdomen of each group. The tumor volume was measured with Vernier caliper every 4 days, and the formula was: volume = (length × width ^2^)/2. The mice were euthanized after 3 weeks. We injected stable transfected cells (1 × 10^6^ cells/100μL PBS) into the tail vein of mice to observe lung metastases. Six weeks later, the nude mice were dissected to observe the lung metastasis.

### Dual Luciferase Reporter Assay

The 3 ‘- UTR sequence of *HHIP* containing mutants (MUT) or wild type (WT) miR - 199b - 5p binding sites was constructed by Genscript (Nanjing, China), and cloned into PGL - 3 luciferase reporting vector. After incubation in 24-well plates for 24 hours, *pGL3 - WT - HHIP or pGL3 - MUT - HHIP 3 ‘- UTR* reporter plasmids were cotransfected with miR -199b-5p mimic or miR-NC with Lipofectamine 3000 (Invitrogen). The luciferase activity of firefly and renal was evaluated by Dual‐Luciferase Assay System (Promega, USA). The relative expression of luciferase activity of firefly was normalized to luciferase activity of Renilla kidney. All procedures were performed in triplicate.

### Statistical Analysis

Each experiment was repeated three times. Difference between two groups were analyzed by t-test, and all data were expressed as mean ± SD. All of the data were analyzed using SPSS17.0 software (SPSS, USA) and were considered to be statistically significant when *p* values were <0.05.

## Results

### Differentially Expressed miRNAS (DE miRNAS) Identification

**Figure 1:**
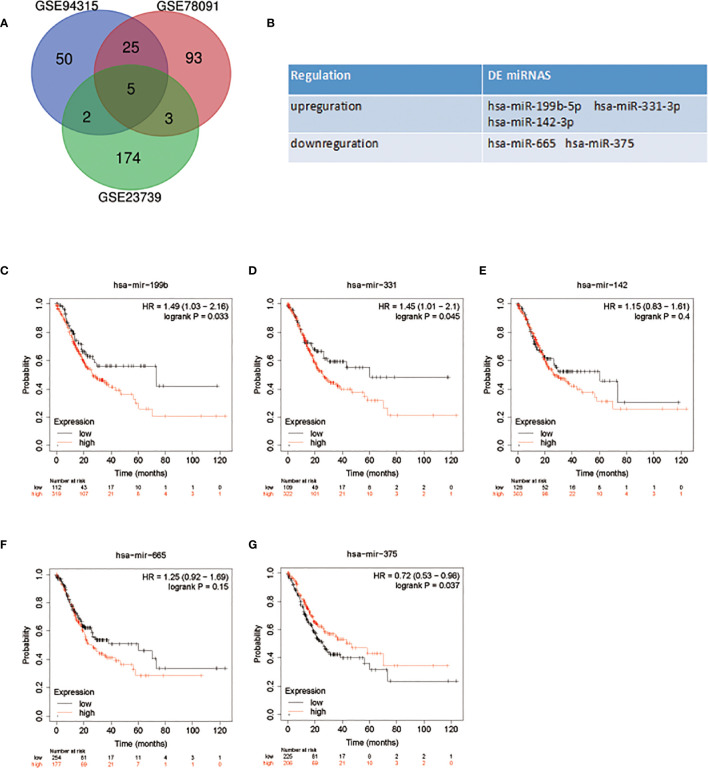
DE miRNAs selection and survival analysis. **(A)** Venn diagram of DE miRNAs in GC. **(B)** The name of DE miRNAs. **(C–G)** Survival analysis of DE miRNAs in patients with GC.

With *P* < 0.05 and [logFC] > 1 as screening conditions, five DE miRNAS were screened by R limma package (**Figure 1A**). MiR-199b-5p, miR-331-3p and miR-142-3p were up-regulated in GC, while miR-665 and miR-375 were down regulated in GC (**Figure 1B**). The specific change thresholds of miRNAs in the three chips are shown in **Table 1**. In order to identify the potential molecules in these miRNAs, we used Kaplan-Meier plotter to analyze the overall survival of all DE miRNAs. The results showed that the high expression of miR-199b and miR-331 was associated with poor prognosis of GC, while the low expression of miR-375 was associated with poor prognosis of GC (**Figure 1C**). However, miR-331-3p and miR-375 have been studied in GC (17, 18). Therefore, we aimed to investigate the related role of miR-199b-5p produced from the 5 ‘arm in GC.

**Table 1:** Differential expression of miRNAs in each dataset.

Genes	GSE78091		GSE93415		GSE23739	
	logFC	*P*.Value	logFC	*P*.Value	logFC	*P*.Value
hsa-miR-199b-5p	1.87	0.001	1.30	0.001	175.52	0.001
hsa-miR-331-3p	1.07	0.001	1.01	0.001	78.45	0.001
hsa-miR-142-3p	1.33	0.002	1.05	0.005	1028.20	0.002
hsa-miR-665	-1.3	0.02	-1.10	0.001	-63.50	0.01
hsa-miR-375	-2.29	0.001	-1.15	0.001	-824.05	0.001

### miR-199b-5p Is Up-Regulated in GC Cells and GC Patients and Promotes GC Cell Proliferation

**Figure 2:**
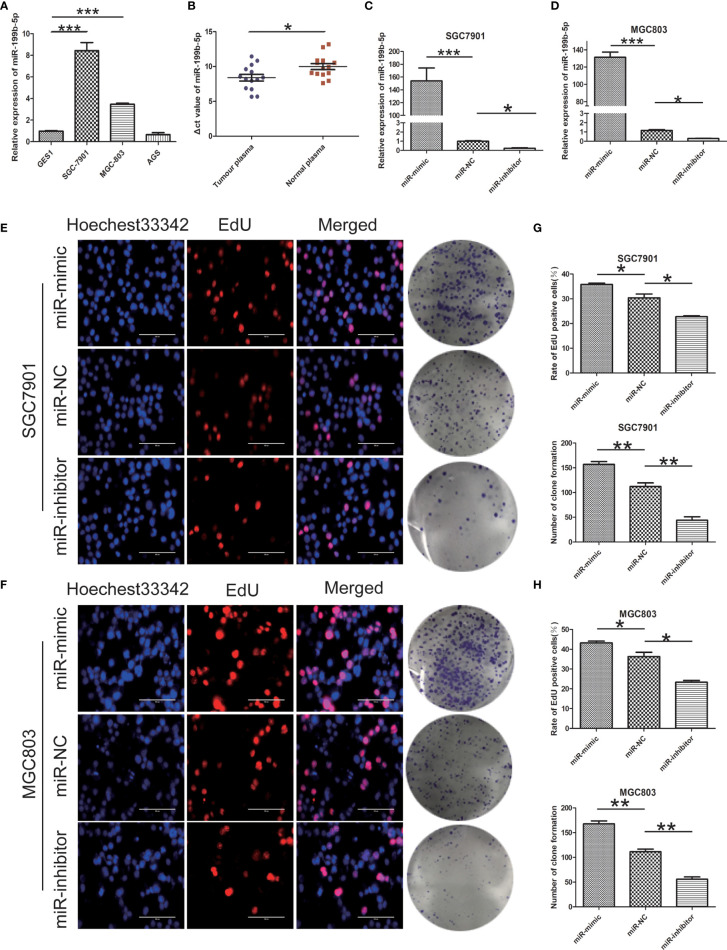
miR - 199b - 5p is upregulated in GC and promotes GC cell proliferation. **(A)** The relative expression of miR-199b-5p in GC cells and GES1. **(B)** Expression of miR-199b-5p in plasma of 14 patients with GC and 14 normal controls. **(C, D)** The relative expression of miR-199b-5p in cells after transfection of miR-199b-5p mimic, NC and inhibitor lentivirus, respectively, in SGC7901 and MGC803. **(E, F)** Representative profiles of EdU assay and colony formation assay in miR-199b-5p mimic and inhibitor groups in SGC7901 and MGC803. **(G, H)** The rate of EdU positive cells and number of colony formation were counted in miR-199b-5p mimic and inhibitor groups. *p < 0.05, **p < 0.01, ***p < 0.001. The data expressed as the mean ± SD.

We detected the expression of miR-199b-5p in normal gastric epithelial cells (GES1) and GC cell lines (SGC7901, MGC803, AGS) by RT-PCR. Compared with GES1, the expression of miR-199b-5p in SGC7901 and MGC803 was significantly higher, which was consistent with GEO database, but there was no difference in the expression of miR-199b-5p in AGS (**Figure 2A**). At the same time, we found that miR-199b-5p was also highly expressed in the plasma of GC patients (**Figure 2B**). To investigate the biological role of miR-199b-5p in GC, we chose SGC7901 and MGC803 for further study. Then miR-199b-5p mimic and inhibitor lentivirus were constructed and transfected into MGC803 and SGC7901 cells respectively. Then RT-PCR was used to verify the transfection efficiency. Compared with the control group, the expression level of miR-199b-5p was significantly increased in the mimic group and decreased in the inhibitor group (**Figures 2C, D**). Edu proliferation assay and clone formation assay were used to detect the proliferation function of miR-199b-5p in GC (**Figures 2E, F**). The results showed that overexpression of miR-199b-5p could promote the proliferation of SGC7901 and MGC803 Cells, while inhibition of miR-199b-5p had the opposite effect (**Figures 2G, H**). In conclusion, the above results showed that overexpression of miR-199b-5p could promote the proliferation of GC cells *in vitro*.

### miR -199b - 5p Enhances Migration and the EMT Processing in GC Cells

**Figure 3:**
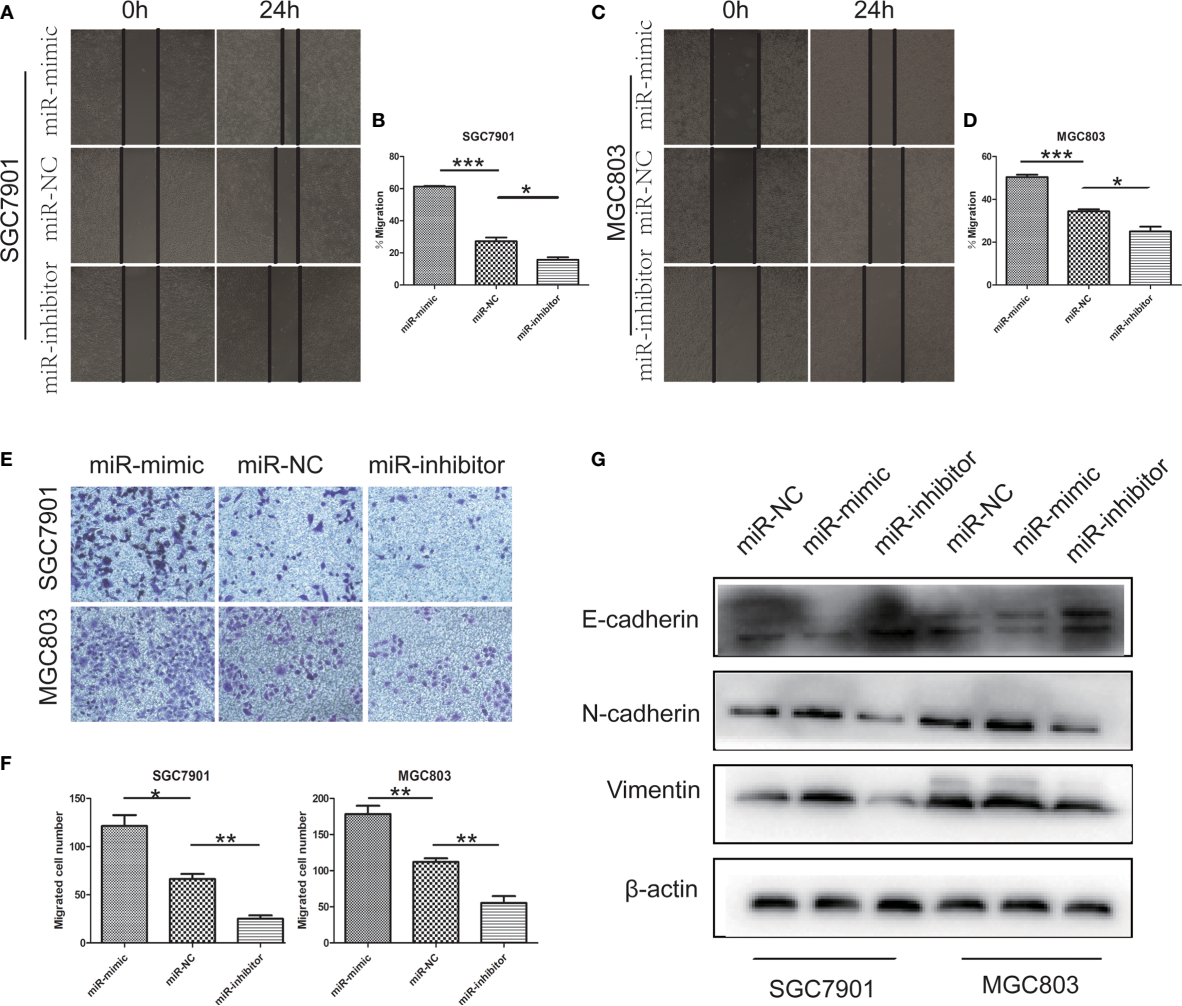
miR -199b - 5p facilitates migration and the EMT processing in GC cells. **(A–D)** Wound-healing assay was used to determine the migration of GC cells after transfection of miR-199b-5p mimic, NC and inhibitor lentivirus, respectively, in SGC7901 and MGC803. **(E, F)** Effects of miR-199b-5p alteration on migration by transwell assay *in vitro*. **(G)** The expression of EMT-associated proteins detected by Western blot when expression of miR-199b-5p was altered in SGC7901 and MGC803. *p < 0.05, **p < 0.01, ***p < 0.001. The data expressed as the mean± SD.

Further study on the effect of miR-199b-5p on GC cells. The effect of miR-199b-5p on the migration of GC cells was detected by wound healing assay and Transwell assay. In wound healing assay, overexpression of miR-199b-5p promoted the migration rate of GC cells. On the contrary, inhibition of miR-199b-5p significantly inhibited the migration of GC cells (**Figures 3A–D**). Consistent with the results of wound healing assay, we found the same results in Transwell assay. The overexpression of miR-199b-5p in SGC7901 and MGC803 Cells promoted the cell migration, while inhibition of miR-199b-5p was lower than that of the control group (**Figures 3E, F**).

In order to clarify whether miR-199b-5p affects the EMT process of GC cells, we detected the EMT related markers by Western blot assay. The analysis showed that the up regulation of miR-199b-5p could reduce the level of E-cadherin and increase the level of vimentin, N-cadherin. However, after inhibiting miR-199b-5p expression, E-cadherin increased in SGC7901 and MGC803, but vimentin and N-cadherin decreased (**Figure 3G**).

These results suggested that miR-199b-5p may play a key role in the EMT process, thus promoting the migration of GC cells *in vitro*.

### miR -199b - 5p Contributes to Tumor Progression and Metastasis *In Vivo*

**Figure 4:**
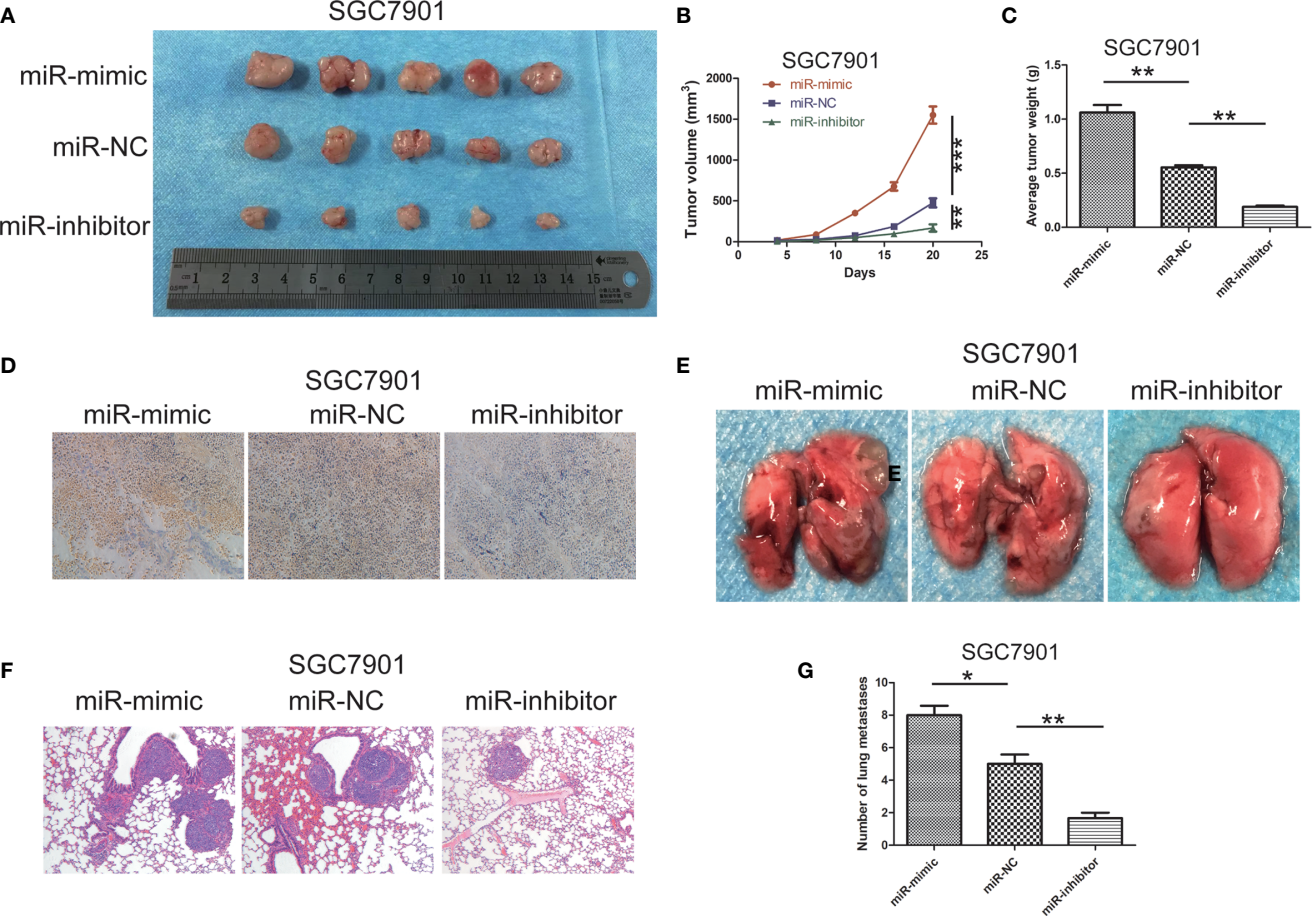
miR -199b - 5p contributes to tumor progression and metastasis *in vivo*. **(A)** photographs of tumors obtained from mice in miR-199b-5p mimic and inhibitor groups. **(B, C)** Tumor volume and weight were calculated in miR-199b-5p mimic and inhibitor groups in SGC7901. volume=(length×width ^2^)/2. **(D)** Ki67 staining assay was used to further verify that miR-199b-5p promoted tumorigenicity. **(E)** Representative images of different groups of lung metastases. **(F)** Representative HE-stained sections of lung from mice in different groups. **(G)** Number of lung metastases in each group. *p < 0.05, **p < 0.01, ***p < 0.001. The data expressed as the mean± SD.

In order to verify the effect of miR-199b-5p on tumor growth *in vivo*, SGC7901 cells stably transfected with miR-199b-5p mimic or miR-199b-5p inhibitors were injected into the flank of nude mice, and SGC7901 cells transfected with miR-NC served as negative control. As shown in **Figures 4A–C**, compared with miR-NC group, the tumor volume and weight in miR-199b-5p mimic group were significantly increased, while those in miR-199b-5p inhibitor group were decreased. The effect of miR-199b-5p on tumorigenesis was further verified by Ki67 staining. The expression of Ki67 in miR-199b-5p mimic group was higher than that in miR-NC group, while the expression of Ki67 in miR-199b-5p inhibitor group was weaker than that in miR-NC group (**Figure 4D**). To further verify the effect of miR-199b-5p on tumor metastasis *in vivo*, stably transfected cells were injected into the tail vein of BALB/C nude mice. After 7 weeks, we found that miR-199b-5p group significantly promoted lung metastasis than miR-NC group. We also found that the miR-199b-5p inhibitor group had a alleviating effect on lung metastasis compared with the miR-NC group (**Figures 4E, G**). Then the lung tissue of mice was stained with H&E assay, and the results were consistent (**Figure 4F**). In conclusion, our results *in vivo* were consistent with those *in vitro*.

### *HHIP* Is a Direct Target of miR- 199b - 5p and Down-Regulated in GC Tissues and Cells

**Figure 5:**
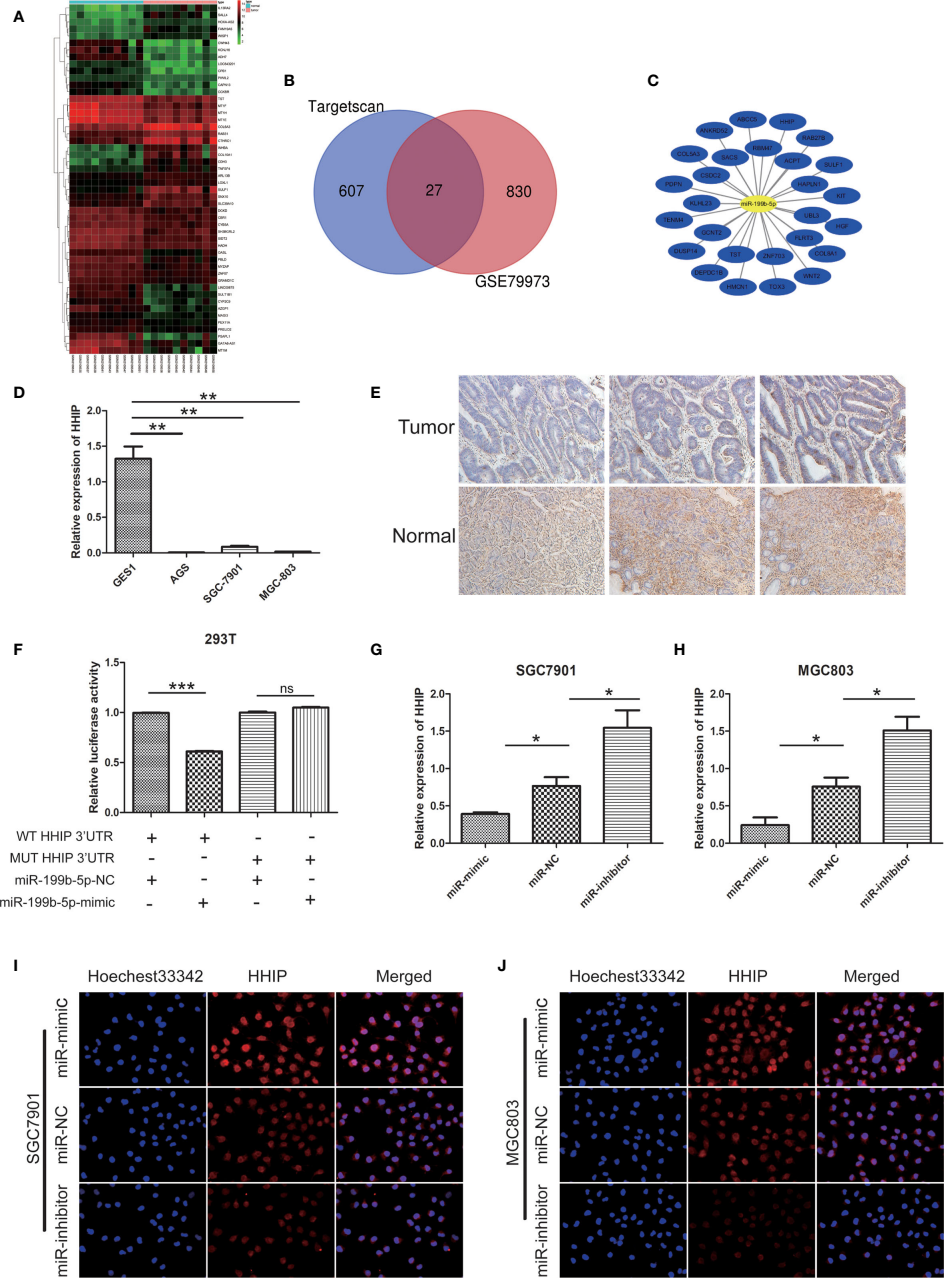
HHIP is a direct target of miR - 199b - 5p and downregulated in GC tissues and cells. **(A)** The heat map of the top 50 genes of DEGs. The vertical axis represents samples. The horizontal axis represents DEGs. **(B)** Venn diagram of DEGs. **(C)** miRNA-DEGs regulatory network. **(D)** The relative expression of miR-199b-5p in GC cells. **(E)** Representative IHC staining of HHIP in five pairs of GC and normal specimens. **(F)** Relative luciferase activity was analyzed in 293T cells co-transfected miR-199b-5p mimics or NC with HHIP-WT or HHIP-MUT, respectively. **(G, H)** The relative expression of HHIP after transfection of miR-199b-5p mimic, NC and inhibitor lentivirus. **(I, J)** The level of HHIP protein was detected by immunofluorescence. *p < 0.05, **p < 0.01, ***p < 0.001. The data expressed as the mean± SD. ns, no significance.

In order to better predict the target gene of miR-199b-5p in GC, we screened the differentially expressed genes(DEGs) in GC tissue through GEO database(**Figure 5A**). Then, the potential target genes of miR-199b-5p were predicted by TargetScan database, and these potential target genes were intersected with the differentially expressed mRNA in GC (**Figure 5B**). The result showed that there were 27 potential target genes with altered expression in GC (**Figure 5C**). *HHIP* was chosen as a candidate gene because it has a potential miR-199b-5p binding site in its 3’UTR, and study have found that *HHIP* plays an anti-cancer role in GC (16).

In our experiments, our results showed that *HHIP* expression in GC cell lines were lower than GES1(**Figure 5D**). The results of immunohistochemistry showed that *HHIP* was mainly expressed in the cytoplasm of GC, and the expression of *HHIP* was lower than that of normal gastric tissue(**Figure 5E**). We further verified whether miR-199b-5p could directly target 3 ‘- UTR of *HHIP* mRNA through Dual luciferase reporter assay. In 293T cells, the *HHIP* 3’UTR sequences of WT and MUT were subcloned into *pGL3* luciferase reporter vector. We noted that Co-transfection of miR-199b-5p mimic and *pGL3-WT*- *HHIP* 3’UTR resulted in decreased luciferase activity compared with the control group. In contrast, overexpression of miR-199b-5p did not affect luciferase activity in *pGL3-MUT*- *HHIP* 3’UTR transfected cells(**Figure 5F**). In addition, we found that overexpression of miR-199b-5p could reduce the mRNA and protein levels of HHIP, but it was the opposite after inhibiting the expression of miR-199b-5p(**Figures 5G–J**). Overall, our results suggested that *HHIP* is a direct target of miR-199b-5p and is frequently down regulated in GC tissues and cells.

### miR -199b - 5p Enhances Proliferation, Migration, and EMT in GC Cells by Targeting HHIP

**Figure 6:**
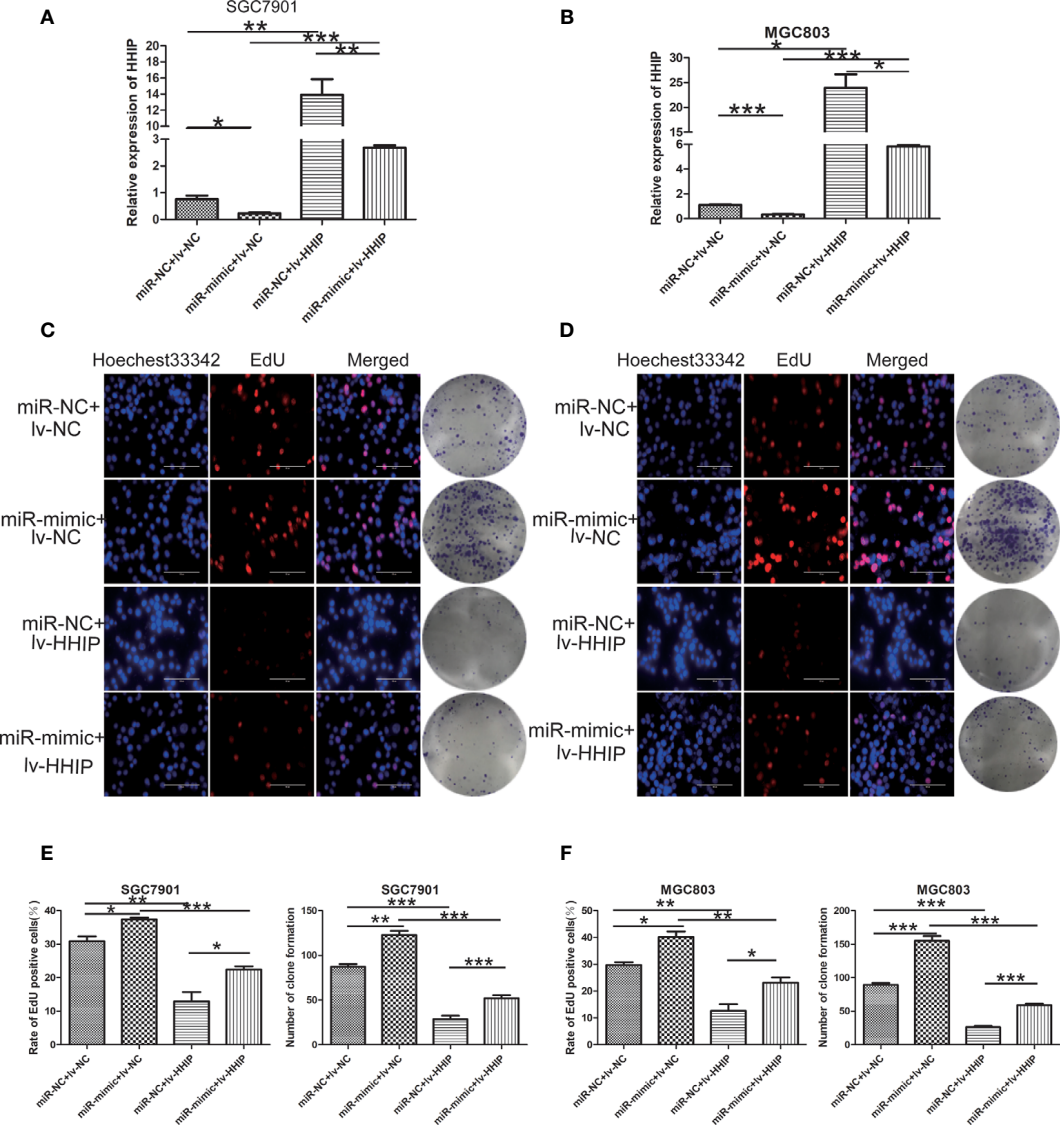
Overexpressed HHIP could partially reverse the effects of miR-199b-5p on GC cells. **(A, B)** RT-PCR was used to verify the expression of HHIP in each group. **(C–F)** EdU incorporation assay and colony formation assay were conducted to verify that ectopic HHIP expression could reverse proliferation induced by miR-199b-5p overexpression in GC cells. *p < 0.05, **p < 0.01, ***p < 0.001. The data expressed as the mean± SD.

**Figure 7:**
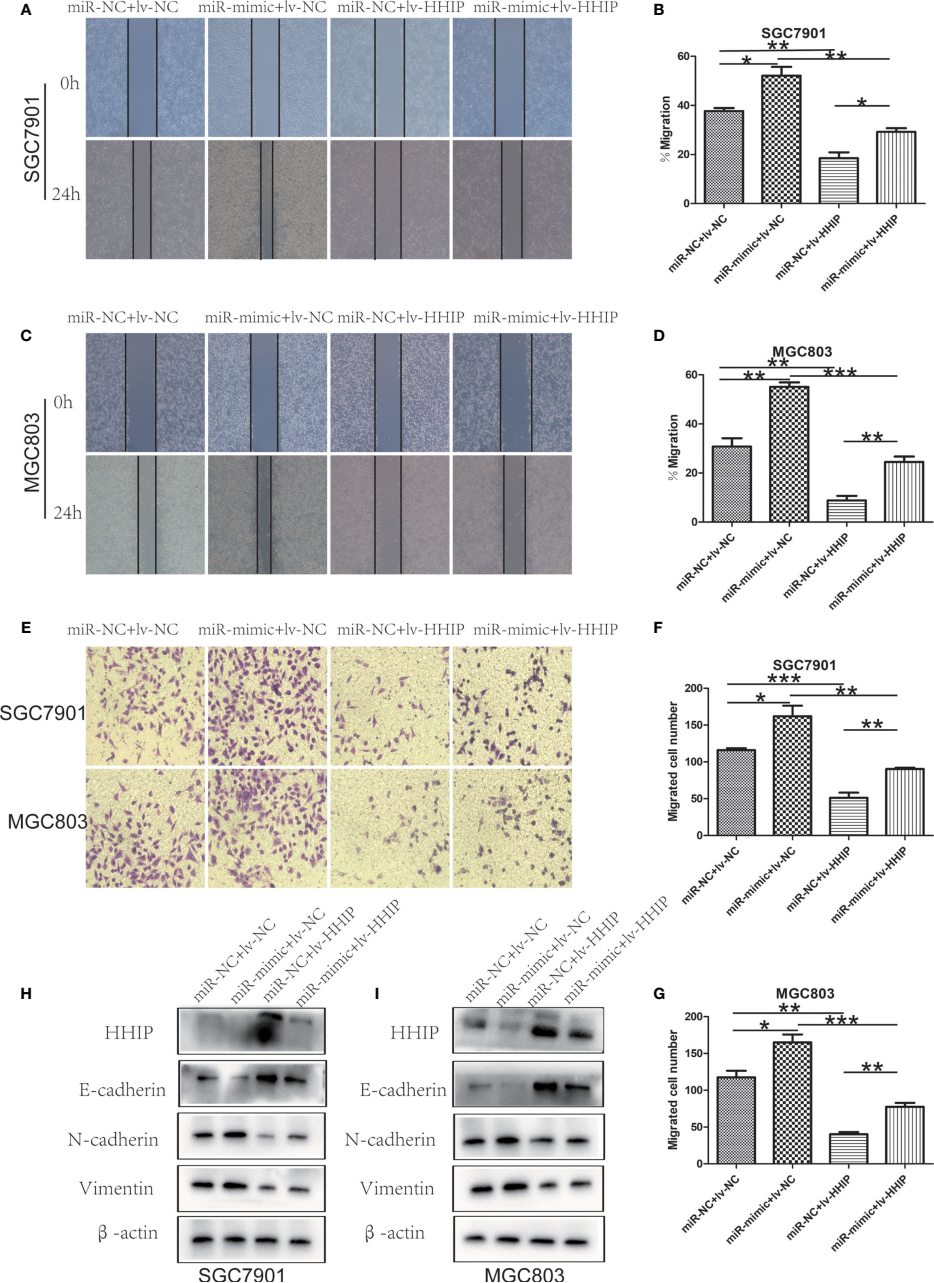
Overexpressed HHIP could partially reverse the effects of miR-199b-5p on GC cells. **(A–D)** The change of cell migration was examined by wound healing assay in SGC7901 and MGC803 cells. **(E–G)** Transwell assay was carried out to confirm the effects of HHIP alteration in migration of GC cells. **(H, I)**, The expression of EMT-associated proteins was detected when HHIP alteration in SGC7901 and MGC803 cells. *p < 0.05, **p < 0.01, ***p < 0.001. The data expressed as the mean± SD.

To further confirm whether mir-199b-5p promotes proliferation, migration and EMT in GC by regulating *HHIP*, we first constructed *lv-HHIP* vector to allow *HHIP* expression. Then, *miR- NC+ lv-NC, miR-mimic + lv-NC, miR-NC + lv-HHIP*, and *miR- mimic + lv-HHIP* were co-transfected into SGC7901 and MGC803 Cells. The expression of *HHIP* was confirmed by RT-PCR and Western blot (**Figures 6A, B**, **7H, I**). Our results show that co-transfection of *lv-HHIP* into miR-199b-5p-mimic cells reversed the proliferation of miR-199b-5p-mimic in EdU assay and clone formation assay(**Figures 6C–F**). Similarly, we found similar results in Transwell assay, wound healing assay and EMT marker detection, that is, overexpression of *HHIP* can partially reversed the migration of miR-199b-5p in GC cells and EMT process(**Figures 7A–I**).These results suggested that miR-199b-5p promoted the proliferation, migration and EMT process of GC cells by directly targeting *HHIP*.

## Discussion

Numerous evidences showed that miRNAs can regulate gene expression by binding to the 3 ‘- untranslated region of downstream genes, and play different roles in cancer progression as carcinogens or tumor suppressors (19–21). Du et al. studies have shown that miR-95 can inhibit GC by regulating dusp5 (22).Li et al. found that miR-20a-5p can promote the progress of GC by inhibiting the expression of WTX (23). Deng et al. also showed that miR-192 and miR-215 could simultaneously target APC and promote the progression of GC (24). Therefore, it is of great significance to explore the biological role of miRNA in the discovery of carcinogenic mechanism and treatment of cancer. It has been found that miR-199b-5p plays a role as a cancer promoting factor in osteosarcoma by regulating cell proliferation, migration, invasion and EMT (10).Li et al. found that miR-199b-5p was highly expressed in cervical cancer and promoted tumor growth and metastasis by down regulating KLK10 (25). At the same time, miR-199b-5p is low expressed in oral cancer cells and promotes oral cancer cell apoptosis (26). However, the potential molecular mechanism of miR-199b-5p in GC remains to be elucidated. In this study, we found that miR-199b-5p was highly expressed in GC through GEO database. Survival analysis also showed that miR-199b was associated with poor prognosis of GC patients. Therefore, we were very interested in the role of miR-199b-5p produced from 5,end arm in GC. Compared with GES1 cells, we found that miR-199b-5p was also highly expressed in SGC7901 and MGC803 Cells. Interestingly, we found that miR-199b-5p was highly expressed in the plasma of GC patients, and we speculated that miR-199b-5p might be an effective molecule for liquid biopsies of GC patients. But this needs to be verified with a large number of samples. Through a series of proliferation and migration experiments, our results showed that overexpression of miR-199b-5p can promote the occurrence of tumor, and the opposite result was obtained after inhibition of miR-199b-5p. *In vivo*, the results are consistent with those *in vitro*. These results suggest that miR-199b-5p may be a potential therapeutic target for GC. It is worth noting that compared with GES1 cells, the expression of miR-199b-5p in AGS cells has no significant difference. It was found that miR-96-5p was significantly up-regulated from liver cirrhosis to dysplastic nodules to advanced liver cancer, but there was no difference in the expression of miR-96-5p between well-differentiated liver cancer and advanced liver cancer (27). We speculate that the expression level of miR-199b-5p may be the same as that of miR-96-5p, and it may be regulated by other potential factors in AGS cells, which needs further study.

To elucidate the mechanism of miR-199b-5p on cell proliferation and migration. We further used bioinformatics analysis to predict the possible target genes of miR-199b-5p in GC cells. Among the candidate target genes, we focused on *HHIP*. *HHIP*, as a member of Hedgehog (*Hh*) family, can compete with *PTCH* gene to bind to *Hh* protein, thus blocking the *Hh* signaling pathway, which has extremely important anti-tumor significance (15, 28, 29). Many studies have shown that *HHIP* plays an anti-tumor role in GC, liver cancer and glioblastoma (16, 30, 31). In our study, compared with GES1 cells, *HHIP* expression was significantly lower in GC cell lines, and our Immunohistochemistry results also suggested that *HHIP* expression was also lower in GC tissues. In addition, Dual luciferase reporter assay confirmed that miR-199b-5p directly combined with *HHIP*, and overexpression of miR-199b-5p could inhibit the expression of *HHIP*. At the same time, overexpression of *HHIP* partially counteracted the effect of miR-199b-5p on cell proliferation and migration. In conclusion, our results show that miR-199b-5p plays a role in GC by down regulating *HHIP*. However, it is worth noting that the expression of HHIP is significantly reduced in AGS cells where miR-199b-5p is not significantly expressed. Studies have shown that HHIP is the target gene of miR-25-3p in hepatocellular carcinoma (32). We speculate that the connection between miR-199b-5p and *HHIP* is regulated by potential factors in AGS cells, but this requires further mechanism research to explore.

EMT refers to the biological process that epithelial cells transform into specific mesenchymal phenotype cells through a specific process (33). In cancer, EMT is associated with tumor metastasis and treatment resistance (34, 35). The typical feature of EMT is that the expression of cell adhesion protein (*E-cadherin*) is decreased, while the expression of interstitial related molecules (*N-cadherin* and *Vimentin*) is increased (36, 37). Therefore, EMT plays an important role in tumor migration and invasion. Recently, more and more studies have confirmed that miRNAs are associated with EMT in malignant tumors. Jaca et al. showed that high expression of miR-21 was associated with *E-cadherin* positive cases (38).Shi et al. also suggested that miR-106a is involved in the progression of oral cancer by regulating *E-cadherin, N-cadherin* and *Vimentin* (39).In this study, we confirmed that overexpression of miR-199b-5p can promote EMT process by significantly reducing the level of *E-cadherin* and up regulating the expression of *N-cadherin* and *Vimentin*. These results were consistent with our *in vivo* and *in vitro* migration results.

With the deepening of miRNA research, they may provide potential and effective choices for clinical diagnosis and treatment of specific malignant tumors (40, 41). According to relevant reports, the drug therapy targeting miRNA has entered the clinical development stage, in which the tumor suppressor miR-34 has entered the phase I clinical trial, and the drug targeting miR-122 has entered the phase II clinical trial for the treatment of hepatitis (42–44). In this study, we found that miR-199b-5p can promote the proliferation, migration and EMT process of GC. It may become a new potential target for the treatment of GC in the future. However, we still need a large number of clinical samples and more related pathways to further study the role of miR-199b-5p in GC.

In short, our data demonstrated that miR-199b-5p is up-regulated in GC. Overexpression of miR-199b-5p could promote GC proliferation, migration and EMT, and this role is played by directly regulating the expression of *HHIP*. In conclusion, our results suggested that miR-199b-5p/*HHIP* pathway axis may be a potential therapeutic target for GC.

## Data Availability Statement

The datasets presented in this study can be found in online repositories. The names of the repository/repositories and accession number(s) can be found in the article/supplementary material.

## Ethics Statement

The studies involving human participants were reviewed and approved by Guangxi Medical University Cancer Hospital Institutional Ethics Committee. The patients/participants provided their written informed consent to participate in this study. The animal study was reviewed and approved by Guangxi Medical University Cancer Hospital Institutional Ethics Committee. Written informed consent was obtained from the owners for the participation of their animals in this study.

## Author Contributions

SC was in charge of the overall experiment. HW, LZ, and MJ were responsible for animal experiments. SW and JL were responsible for the collection of experimental specimens. AL was responsible for the design and management of the experiment. All authors contributed to the article and approved the submitted version.

## Funding

This project was supported by Guangxi Natural Science Foundation(No. 2017GXNSFAA198065),Guangxi Medical High-level Backbone Talent “139” Plan (No. G201903015), Guangxi Key R & D Plan (AB18221084),and Funding for the development and promotion of suitable medical and health technologies in Guangxi (S2018059).

## Conflict of Interest

The authors declare that the research was conducted in the absence of any commercial or financial relationships that could be construed as a potential conflict of interest.

## Publisher’s Note

All claims expressed in this article are solely those of the authors and do not necessarily represent those of their affiliated organizations, or those of the publisher, the editors and the reviewers. Any product that may be evaluated in this article, or claim that may be made by its manufacturer, is not guaranteed or endorsed by the publisher.

## References

[1] Sung H Ferlay J Siegel RL Laversanne M Soerjomataram I Jemal A. Global Cancer Statistics 2020: GLOBOCAN Estimates of Incidence and Mortality Worldwide for 36 Cancers in 185 Countries. CA Cancer J Clin (2021) 71(3):209–49. 10.3322/caac.21660 33538338

[2] Chen W Zheng R Baade PD Zhang S Zeng H Bray F. Cancer Statistics in China, 2015. CA Cancer J Clin (2016) 66(2):115–32. 10.3322/caac.21338 26808342

[3] Smyth EC Nilsson M Grabsch HI van Grieken NCT Lordick F. Gastric Cancer. Lancet (2020) 396(10251):635–48. 10.1016/S0140-6736(20)31288-5 32861308

[4] Ikoma N Blum M Chiang YJ Estrella JS Chowdhuri SR Foumier K. Race Is a Risk for Lymph Node Metastasis in Patients With Gastric Cancer. Ann Surg Oncol (2017) 24(4):960–5. 10.1245/s10434-016-5645-x 27778127

[5] Wang B You Z Ren D. Target-Assisted FRET Signal Amplification for Ultrasensitive Detection of microRNA. Anal (2019) 144(7):2304–11. 10.1039/C8AN02266F 30672513

[6] Zhao C Zhang Y Popel AS. Mechanistic Computational Models of MicroRNA-Mediated Signaling Networks in Human Diseases. Int J Mol Sci (2019) 20(2):421. 10.3390/ijms20020421 PMC6358731 30669429

[7] Lee RC Feinbaum RL Ambros V. The C. Elegans Heterochronic Gene Lin-4 Encodes Small RNAs With Antisense Complementarity to Lin-14. Cell (1993) 75(5):843–54. 10.1016/0092-8674(93)90529-Y 8252621

[8] Rodriguez A Griffiths-Jones S Ashurst JL Bradley A. Identification of Mammalian microRNA Host Genes and Transcription Units. Genome Res (2004) 14(10a):1902–10. 10.1101/gr.2722704 PMC524413 15364901

[9] Yu J Peng J Luan Z. MicroRNAs as a Novel Tool in the Diagnosis of Liver Lipid Dysregulation and Fatty Liver Disease. Molecules (2019) 24(2):230. 10.3390/molecules24020230 PMC6358728 30634538

[10] Chen Z Zhao G Zhang Y Ma Y Ding Y Xu N. MiR-199b-5p Promotes Malignant Progression of Osteosarcoma by Regulating HER2. J buon (2018) 23(6):1816–24. 30610808

[11] Zhao Z Zhao S Luo L Xange Q Zhu Z Wang J. miR-199b-5p-DDR1-ERK Signalling Axis Suppresses Prostate Cancer Metastasis *via* Inhibiting Epithelial-Mesenchymal Transition. Br J Cancer (2021) 124(5):982–94. 10.1038/s41416-020-01187-8 PMC7921430 33239676

[12] Lin X Qiu W Xiao Y Ma J Xu F Zhang K. MiR-199b-5p Suppresses Tumor Angiogenesis Mediated by Vascular Endothelial Cells in Breast Cancer by Targeting Alk1. Front Genet (2019) 10:1397. 10.3389/fgene.2019.01397 32082362 PMC7002562

[13] Ingham PW McMahon AP. Hedgehog Signaling in Animal Development: Paradigms and Principles. Genes Dev (2001) 15(23):3059–87. 10.1101/gad.938601 11731473

[14] Zhao JG Wang JF Feng JF Jin XY Ye WL. HHIP Overexpression Inhibits the Proliferation, Migration and Invasion of Non-Small Cell Lung Cancer. PloS One (2019) 14(11):e0225755. 10.1371/journal.pone.0225755 31765425 PMC6876884

[15] Chang L Zhang P Zhao D Liu H Wang Q Li C. The Hedgehog Antagonist HHIP as a Favorable Prognosticator in Glioblastoma. Tumour Biol (2016) 37(3):3979–86. 10.1007/s13277-015-3442-y 26482617

[16] Sun H Ni SJ Ye M Weng W Zhang Q Zhang M. Hedgehog Interacting Protein 1 Is a Prognostic Marker and Suppresses Cell Metastasis in Gastric Cancer. J Cancer (2018) 9(24):4642–9. 10.7150/jca.27686 PMC6299386 30588248

[17] Guo X Guo L Ji J Zhang J Zhang J Chen X. miRNA-331-3p Directly Targets E2F1 and Induces Growth Arrest in Human Gastric Cancer. Biochem Biophys Res Commun (2010) 398(1):1–6. 10.1016/j.bbrc.2010.05.082 20510161

[18] Shen ZY Zhang ZZ Liu H Zhao EH Cao H. miR-375 Inhibits the Proliferation of Gastric Cancer Cells by Repressing ERBB2 Expression. Exp Ther Med (2014) 7(6):1757–61. 10.3892/etm.2014.1627 PMC4043572 24926380

[19] Yang YB Tan H Wang Q. MiRNA-300 Suppresses Proliferation, Migration and Invasion of Non-Small Cell Lung Cancer *via* Targeting ETS1. Eur Rev Med Pharmacol Sci (2019) 23(24):10827–34. 10.26355/eurrev_201912_19786 31858551

[20] Yu Y Wang Y Xiao X Cheng W Hu L Yao W. MiR-204 Inhibits Hepatocellular Cancer Drug Resistance and Metastasis Through Targeting NUAK1. Biochem Cell Biol = Biochimie Biol Cell (2019) 97(5):563–70. 10.1139/bcb-2018-0354 30807203

[21] Zhang W Liao K Liu D. MiRNA-12129 Suppresses Cell Proliferation and Block Cell Cycle Progression by Targeting SIRT1 in GASTRIC Cancer. Technol Cancer Res Treat (2020) 19:1533033820928144. 10.1177/1533033820928144 32508267 PMC7281879

[22] Du M Zhuang Y Tan P Yu Z Zhang X Wang A. microRNA-95 Knockdown Inhibits Epithelial-Mesenchymal Transition and Cancer Stem Cell Phenotype in Gastric Cancer Cells Through MAPK Pathway by Upregulating DUSP5. J Cell Physiol (2020) 235(2):944–56. 10.1002/jcp.29010 31309567

[23] Li J Ye D Shen P Liu X Zhou P Zhu G. Mir-20a-5p Induced WTX Deficiency Promotes Gastric Cancer Progressions Through Regulating PI3K/AKT Signaling Pathway. J Exp Clin Cancer Res: CR (2020) 39(1):212. 10.1186/s13046-020-01718-4 33032635 PMC7545863

[24] Deng S Zhang X Qin Y Chen W Fan H Feng X. miRNA-192 and -215 Activate Wnt/β-Catenin Signaling Pathway in Gastric Cancer *via* APC. J Cell Physiol (2020) 235(9):6218–29. 10.1002/jcp.29550 32091625

[25] Xu LJ Duan Y Wang P Yin HQ. MiR-199b-5p Promotes Tumor Growth and Metastasis in Cervical Cancer by Down-Regulating KLK10. Biochem Biophys Res Commun (2018) 503(2):556–63. 10.1016/j.bbrc.2018.05.165 29807015

[26] Wang H Guo Y Mi N Zhou L. miR-101-3p and miR-199b-5p Promote Cell Apoptosis in Oral Cancer by Targeting BICC1. Mol Cell Probes (2020) 52:101567. 10.1016/j.mcp.2020.101567 32259627

[27] Zhang H Xing AY Ma RR Wang YW Liu YH Gao P. Diagnostic Value of miRNA-96-5p/3p in Dysplastic Nodules and Well-Differentiated Small Hepatocellular Carcinoma. Hepatol Res (2016) 46(8):784–93. 10.1111/hepr.12628 26609665

[28] Büller NV Rosekrans SL Westerlund J van den Brink GR. Hedgehog Signaling and Maintenance of Homeostasis in the Intestinal Epithelium. Physiol (Bethesda Md) (2012) 27(3):148–55. 10.1152/physiol.00003.2012 22689790

[29] Wei H Li J Shi S Zhang L Xiang A Shi X. Hhip Inhibits Proliferation and Promotes Differentiation of Adipocytes Through Suppressing Hedgehog Signaling Pathway. Biochem Biophys Res Commun (2019) 514(1):148–56. 10.1016/j.bbrc.2019.04.047 31027733

[30] Wang X Ma W Yin J Chen M Jin H. HHIP Gene Overexpression Inhibits the Growth, Migration and Invasion of Human Liver Cancer Cells. J Buon (2020) 25(5):2424–9. 33277865

[31] Shahi MH Zazpe I Afzal M Sinha S Rebhun RB Melendez B. Epigenetic Regulation of Human Hedgehog Interacting Protein in Glioma Cell Lines and Primary Tumor Samples. Tumour Biol (2015) 36(4):2383–91. 10.1007/s13277-014-2846-4 PMC5012430 25416442

[32] Ouyang Y Tang Y Fu L Peng S Wu W Tan D. Exosomes Secreted by Chronic Hepatitis B Patients With PNALT and Liver Inflammation Grade ≥ A2 Promoted the Progression of Liver Cancer by Transferring miR-25-3p to Inhibit the Co-Expression of TCF21 and HHIP. Cell Proliferation (2020) 53(7):e12833. 10.1111/cpr.12833 32525231 PMC7377934

[33] Saitoh M. Involvement of Partial EMT in Cancer Progression. J Biochem (2018) 164(4):257–64. 10.1093/jb/mvy047 29726955

[34] Cho ES Kang HE Kim NH Yook JI. Therapeutic Implications of Cancer Epithelial-Mesenchymal Transition (EMT). Arch Pharmacal Res (2019) 42(1):14–24. 10.1007/s12272-018-01108-7 30649699

[35] Yeung KT Yang J. Epithelial-Mesenchymal Transition in Tumor Metastasis. Mol Oncol (2017) 11(1):28–39. 10.1002/1878-0261.12017 28085222 PMC5242415

[36] Wang L Li B Zhang L Li Q He Z Huang X. miR-664a-3p Functions as an Oncogene by Targeting Hippo Pathway in the Development of Gastric Cancer. Cell Proliferation (2019) 52(3):e12567. 10.1111/cpr.12567 30883979 PMC6536452

[37] Lei H Gao Y Xu X. LncRNA TUG1 Influences Papillary Thyroid Cancer Cell Proliferation, Migration and EMT Formation Through Targeting miR-145. Acta Biochim Biophys Sin (2017) 49(7):588–97. 10.1093/abbs/gmx047 28645161

[38] Jaca A Govender P Locketz M. The Role of miRNA-21 and Epithelial Mesenchymal Transition (EMT) Process in Colorectal Cancer. J Clin Pathol (2017) 70(4):331–56. 10.1136/jclinpath-2016-204031 27672217

[39] Shi B Ma C Liu G Guo Y. MiR-106a Directly Targets LIMK1 to Inhibit Proliferation and EMT of Oral Carcinoma Cells. Cell Mol Biol Lett (2019) 24:1. 10.1186/s11658-018-0127-8 30873211 PMC6402160

[40] Mishra S Yadav T Rani V. Exploring miRNA Based Approaches in Cancer Diagnostics and Therapeutics. Crit Rev Oncol/Hematol (2016) 98:12–23. 10.1016/j.critrevonc.2015.10.003 26481951

[41] Link A Kupcinskas J. MicroRNAs as non-Invasive Diagnostic Biomarkers for Gastric Cancer: Current Insights and Future Perspectives. World J Gastroenterol (2018) 24(30):3313–29. 10.3748/wjg.v24.i30.3313 PMC6092583 30122873

[42] Rupaimoole R Slack FJ. MicroRNA Therapeutics: Towards a New Era for the Management of Cancer and Other Diseases. Nat Rev Drug Discov (2017) 16(3):203–22. 10.1038/nrd.2016.246 28209991

[43] Gibson NW. Engineered microRNA Therapeutics. J R Coll Phys Edinburgh (2014) 44(3):196–200. 10.4997/JRCPE.2014.302 25318394

[44] Zhang L Liao Y Tang L. MicroRNA-34 Family: A Potential Tumor Suppressor and Therapeutic Candidate in Cancer. J Exp Clin Cancer Res: CR (2019) 38(1):53. 10.1186/s13046-019-1059-5 30717802 PMC6360685

